# A system for studying evolution of life-like virtual organisms

**DOI:** 10.1186/1745-6150-1-23

**Published:** 2006-08-17

**Authors:** Alex A Neyfakh, Natalya N Baranova, Lev J Mizrokhi

**Affiliations:** 1 Center for Pharmaceutical Biotechnology, University of Illinois at Chicago, Chicago, IL 60607, USA; 2 Private individual, New York, NY 10001, USA

## Abstract

**Background:**

Fitness landscapes, the dependences of fitness on the genotype, are of critical importance for the evolution of living beings. Unfortunately, fitness landscapes that are relevant to the evolution of complex biological functions are very poorly known. As a result, the existing theory of evolution is mostly based on postulated fitness landscapes, which diminishes its usefulness. Attempts to deduce fitness landscapes from models of actual biological processes led, so far, to only limited success.

**Results:**

We present a model system for studying the evolution of biological function, which makes it possible to attribute fitness to genotypes in a natural way. The system mimics a very simple cell and takes into account the basic properties of gene regulation and enzyme kinetics. A virtual cell contains only two small molecules, an organic nutrient A and an energy carrier X, and proteins of five types – two transcription factors, two enzymes, and a membrane transporter. The metabolism of the cell consists of importing A from the environment and utilizing it in order to produce X and an unspecified end product. The genome may carry an arbitrary number of genes, each one encoding a protein of one of the five types. Both major mutations that affect whole genes and minor mutations that affect individual characteristics of genes are possible. Fitness is determined by the ability of the cell to maintain homeostasis when its environment changes. The system has been implemented as a computer program, and several numerical experiments have been performed on it. Evolution of the virtual cells usually involves a rapid initial increase of fitness, which eventually slows down, until a fitness plateau is reached. The origin of a wide variety of genetic networks is routinely observed in independent experiments performed under the same conditions. These networks can have different, including very high, levels of complexity and often include large numbers of non-essential genes.

**Conclusion:**

The described system displays a rich repertoire of biologically sensible behaviors and, thus, can be useful for investigating a number of unresolved issues in evolutionary biology, including evolution of complexity, modularity and redundancy, as well as for studying the general properties of genotype-to-fitness maps.

**Reviewers:**

This article was reviewed by Drs. Eugene Koonin, Shamil Sunyaev and Arcady Mushegian.

## Open peer review

Reviewed by Drs. Eugene Koonin, Shamil Sunyaev and Arcady Mushegian. For the full reviews, please go to the Reviewers' comments section.

## Background

Among the fields of biology, evolutionary biology enjoys one of the highest levels of theoretical sophistication. However, theoretical treatments of evolution are mostly confined to the level of populations, where organisms are represented as black boxes and, thus, the genotype-to-fitness maps (fitness landscapes) must be simply postulated, because they cannot be inferred. Population-level theory has been very successful in studying the dynamics of genotype frequencies [1] but can never address the key issues concerned with evolution of functioning phenotypes, such as optimality, complexity, modularity, robustness, and evolve-ability. Unfortunately, organism-level theory of evolution, which does not ignore the internal workings of organisms and derives the fitness of a genotype from an explicit model of functioning of the corresponding phenotype, is still in its infancy.

Most of organism-level theoretical analyses of evolution have been performed on "organisms" which hardly represent real life as it is known to laymen or, for that matter, to biologists. Instead of models of cells or of multicellular organisms, these analyses deal with mathematical algorithms. Computer scientists, impressed by the beauty and apparent universality of Darwin's theory, proposed a concept of genetic algorithm, a software environment in which strings of information ("chromosomes") undergo repetitive cycles of fitness determination, selection, and mutation/recombination [2,3]. Gradually, the strings evolve and become increasingly fit to the goal set by the experimenter. Genetic algorithms proved to be efficient in diverse optimization tasks in engineering [4,5] and software design [6].

This methodology, together with some other achievements such as theory of automata, led to creation of what became known as "artificial life" (see ref. 7 for review). Virtual organisms which constitute artificial life ("Life", "Polyworld", "Tierra", "Avida", etc.) consist of pieces of program code that either compete for computer memory or try to outperform each other in logical operations or mathematical calculations. Thus, definitions of fitness in artificial life experiments are usually biologically meaningless. Such experiments firmly demonstrated the possibility of creating evolving entities in the computer, but it is probably fair to say that their impact on biology has so far been rather limited. Even the potential importance of artificial life for mainstream evolutionary biology [8-11] is not obvious, due to profound differences between artificial and real life.

In contrast, there are only a handful of organism-level models of evolution which are inspired by real biochemical and physiological processes. In particular, a system of mutually regulating virtual DNA-binding proteins was shown to evolve certain patterns in its dynamic, for example to form a bi-stable switch resembling the cI-cro switch that controls lysogenic state of bacteriophage λ [12]. In [13] the authors demonstrated the possibility to evolve an enzymatic cascade capable of performing a mathematical operation, such as producing a reaction product in the amount equal to the cubic root of the amount of the reaction substrate.

The time is clearly ripe for more work of this kind. Mathematical and computational models of real biological phenomena, such as interactions of proteins in regulatory networks (see, for example, ref. 14) or fluxes of organic substances in well-characterized metabolic pathways (reviewed in ref. 15), are becoming more and more successful. Models of biochemical networks in microorganisms became trustable enough to make computational predictions about the paths of evolutionary adaptation of these networks to various sources of energy, carbon, nitrogen, etc. [16]. However, these predictions are based not on explicit modeling of evolution, but on constraints that the structure of the network and the nature of its components impose on possible evolutionary paths. This approach depends on a reasonable assumption that evolution would be able to optimize the biochemistry in any way that does not violate these constraints. Such modeling studies are very instructive from a biochemical perspective and will likely have a strong impact on biotechnology, but they tell us little about evolution of life *per se*.

Here we propose a system for studying evolution of virtual unicellular organisms. This system is based on a rather simple model of a cell, which, nevertheless, includes intracellular processes at all levels. Numerous evolutionary experiments with this system revealed a rich variety of behaviors. Some of the observations we made can shed light on the evolution of complexity and on the origin of non-essential genes. Further investigations of this, and similar, systems can help to elucidate a wide variety of issues concerned with the evolution of life.

## Results

### A system for modelling evolution of a very simple cell

Our general approach is to consider unicellular virtual organisms with a simple structure, which resembles the structure of only a small subsystem of a real cell. Nevertheless, processes along the whole path from genotype to phenotype to fitness are present in these model cells. We explicitly model inheritance with mutation, genes and regulation of their expression, gene-to-gene interactions mediated by DNA-protein interactions, enzymatic catalysis, cellular homeostasis, membrane transport, and protein degradation. Let us describe how all these processes are represented in our system.

#### Informal overview

**Figure 1:**
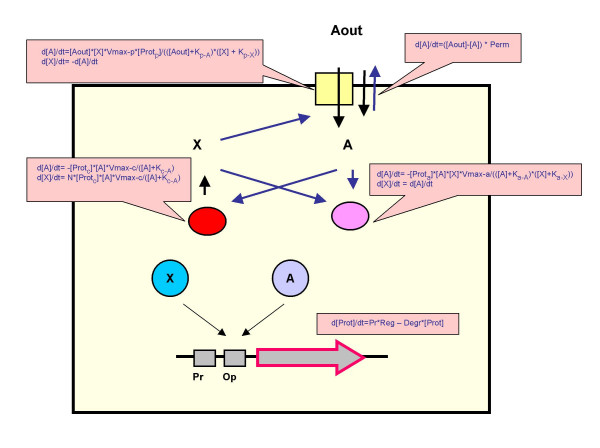
**Scheme of processes in the model organism**. A gene, proteins of five different types and all the reactions that involve molecules A and X are shown (see text). Insets show differential equations which describe fluxes of A and X and of the proteins.

Major features of the virtual unicellular organisms that we designed (from now on we will omit the word "virtual") are shown in Fig. 1. The properties of an organism are determined by its genome, which consists of a set of genes. Each gene is a collection of characteristics: the type of the encoded protein (a pump, an enzyme of either of the two types, or a transcription factor of either of the two types), the promoter strength, the operator type, and several numerical constants which describe the encoded protein. In particular, if the protein is a transcription factor (TF), it has a type, which determines the type(s) of operators it can bind.

The rate of synthesis of a protein encoded by the gene is determined by the strength of its promoter and the effects of TFs (circles in Fig. 1) binding its operator. A TF is modulated by the binding of a ligand, which is either molecule A (light blue circle) or molecule X (dark blue circle), and the strength of this binding is determined by the corresponding constant of dissociation. The effect of a TF on transcription is described by two constants, EffApo (the effect of a ligand-free apo-form of the TF) and EffBound (the effect of a TF bound to the ligand). The EffApo constant determines whether the ligand-free TF is a repressor (EffApo < 1) or an activator (EffApo > 1) of transcription. The ligand can either stimulate (EffBound > EffApo) or inhibit (EffBound < EffApo) transcription of the regulated gene. Posttranscriptional regulation of gene expression is not modeled.

An organism has a very simple metabolism: molecule A, which serves as a source of both energy and carbon, is imported and an energy carrier molecule X is synthesised. For example, A can represent glucose and X can represent ATP in real organisms. A can diffuse in and out of an organism at a low rate, and can also be pumped into the organism by protein pumps (yellow square) that use energy stored in X. Metabolism involves enzymes of two types. Enzymes of the first type (red ellipse) uses energy stored in one molecule of A to synthesize several molecules of X. Enzymes of the second type (magenta ellipse) consume both A and X to synthesize other, unspecified, organic molecules. Pumps and enzymes follow Michaelis-Menten kinetics and the fluxes of A and X through each of these proteins are therefore described by differential equations which contain the protein-specific constants, K_MA_, K_MX _and V_max_, and the concentration of the protein.

Organisms form populations of moderate size and are subject to mutation and selection. Initially, we attempted to select our organisms for the most efficient production of biomass, by maximizing the product of concentrations of A and X. This, however, yielded rather trivial results: the pumps and enzymes quickly evolved the lowest possible values of K_M _and the highest possible values of V_max_. More interesting results were obtained when fitness of an organism was determined by its ability to maintain homeostasis. Let us now describe the system more formally.

#### Level of genotypes

##### 1) Genome

An organism can possess an arbitrary number of genes. The order of these genes on one or many chromosomes, either linear or circular, can be specified.

##### 2) Mutation

Both point mutations and major mutations are possible. A point mutation changes one of the parameters which describe an individual gene. A major mutation affects complete gene(s) and can be a duplication, deletion, insertion, or reversible inactivation of one gene or a block of successive genes on a chromosome.

##### 3) Reproduction

Reproduction can be either asexual or sexual, in which case one of several possible kinds of genetic exchange between organisms take place.

##### 4) An individual gene

A gene consists of the following components: one promoter Pr, one operator Op (a binding site with which transcription factors can interact), and a protein-coding region. Orientation of a gene on the chromosome is not specified and, generally, the nucleotide sequences are not considered explicitly. Instead, a gene directly encodes the following parameters:

(a) A promoter is described by one parameter, its strength Pst, which is a non-negative real number. Pst determines the rate of transcription of the gene, which can also be affected by operator-mediated regulation.

(b) An operator is described by one parameter, its type Ty, which is an integer number determining which transcription factors can bind the operator. There is no "strength" parameter corresponding to an operator.

(c) A protein-coding region is described by the following parameters:

(i) The type of the encoded protein. Currently, there are five possible protein types.

(ii) The properties of the encoded protein, described by the protein type-specific set of parameters (see below).

#### Level of phenotypes

##### 5) Small molecules

There are two small molecules, A ("glucose") and X ("ATP").

##### 6) Protein types and cellular processes

There are five biological activities in our organisms, each one mediated by proteins of the corresponding type. The first two types of proteins are TFs, which regulate gene expression, and the remaining three types of proteins constitute the metabolism (Fig. 1). A protein of each type is described by the corresponding set of parameters, determined by the gene which encodes the protein. A genome can contain an arbitrary number of genes, each with its own set of parameters, all encoding proteins of one particular type. These types are characterized as follows:

(a) A-responsive TF (TF-A) can regulate expression of genes, has molecule A as its ligand, and is characterized by five parameters:

(i) Type, an integer number. TF-A interacts only with operators having the matching (exactly or approximately) type(s).

(ii) K_d_, constant of dissociation, inverse concentration at which 50% of the ligand is bound, which describes how TF-A binds its ligand A,

(iii) K_b_, binding constant, inverse concentration of the TF at which 50% the available binding sites are bound, which describes how TF-A binds operators of the matching type(s). Binding to operators is not affected by the ligand.

(iv) An effect constant EffApo-A, which describes the effect of a ligand-free TF-A on transcription.

(v) Another effect constant EffBound-A, which describes the effect of a ligand-bound TF-A on transcription.

(b) X-responsive TF (TF-X) has analogous properties, except that X, and not A, is the ligand.

(c) A-pump imports molecule A into the cell, using energy stored in X. An A-pump is characterized by two Michaelis constants, K_p-A _and K_p-X_, for outside A and for inside X, respectively, as well as by V_max-P_, the maximal rate of A import.

(d) A → X (catabolism) enzyme which synthesizes X from A, is characterized by Michaelis constant K_c-A _for A and by the maximal rate V_max-c_, but has no Michaelis constant for X, because the reaction is assumed to be irreversible.

(e) A,X → product (anabolism) enzyme which synthesizes an unspecified product from A and X, is characterized by Michaelis constants for A and X, K_a-A_, and K_a-X_, and by the maximal rate V_max-a_. The reaction is assumed to be irreversible.

We also consider two processes which are not driven by proteins:

f) Passive import and export of A through the cell membrane is described by the rate Perm.

g) Protein degradation is described by rate Degr, the same for proteins of all types.

##### 7) Dynamics of the cell

At a particular moment of time, the cell is described by the following dynamical variables: concentrations of the two small molecules, [A] and [X], and concentrations of the product of each of its genes. Thus, we do not model any internal structure of the cell. The following processes are considered.

a) Binding of a TF to a ligand. We assume that this process is much faster than changes in concentrations of proteins and of small molecules. Thus, we use the framework of fast-slow dynamics [17] and do not consider this process explicitly. Instead, we assume that W, the fraction of the TF molecules bound to its ligand, is simply the function of the current concentration of the ligand:

W = [ligand] × K_d_/(1+ [ligand] × K_d_)     (1)

b) Binding of a TF to an operator. Again, we assume that this process is very fast, so that V, the fraction of time during which a particular operator is bound by a particular TF, in either apo or ligand-bound form, is simply the function of the current concentration of all the TFs which can bind this operator.

V = [TF in a particular form] × K_b_/binding polynomial     (2)

where binding polynomial is 1 plus the sum of [TF in a particular form] × K_b _terms for all TFs which can bind the operator. We neglect small declines in concentrations of TFs due to their binding to operators.

c) Changes in protein concentrations. The dynamics of [Prot], the concentration of the protein encoded by a particular gene, are described by the corresponding differential equation in Fig. 1, in which the first term on the right-hand side describes protein synthesis and the second term describes protein degradation. The multiplier Reg describes the impact of operator-mediated regulation of transcription and is given by

Reg = Σ V_i _× E_i _    (3)

where V_i _is the fraction of time the operator is in a particular state (i. e., unbound or bound by a particular TF, in either apo or ligand-bound form; Σ V_i _= 1), and E_i _is the effect of the corresponding state on the rate of transcription (*i. e*., 1 or either EffApo or EffBound, for the corresponding TFs).

d) Active intake of molecule A. The dynamics of [A] and [X] due to import of molecule A by A-pump protein encoded by one particular type of a gene are described by the corresponding differential equations in Fig. 1. The total active intake of A (and the corresponding expenditure of X,) is the sum of contributions of all the pump proteins available in the cell, which are generally characterized by different values of V_max-p_, K_p-A_, and K_p-X_.

e) Passive diffusion of A through the membrane. The dynamics of [A] due to this process are described by the corresponding differential equation in Fig. 1.

f) Synthesis of molecule X. The dynamics of [X] and [A] due to action of the catabolism enzyme encoded by one particular gene are described by the corresponding differential equations in Fig. 1. The total rate of synthesis of X, and of the corresponding expenditure of A, is the sum of contributions of all the catabolism enzyme proteins available in the cell.

g) Consumption of molecules A and X for anabolism. The dynamics of [A] and [X] due to action of the anabolism enzyme encoded by one particular gene are described by the corresponding differential equations in Fig. 1. The total rate of A and X consumptions is the sum of contributions of all the anabolism enzyme proteins available in the cell.

h) Changes of concentrations of molecules A and X. The dynamics of [A] and [X] are described by differential equations with right-hand sides which are sums of all the relevant contributions:

d [A]/dt = Active intake + Passive transport + Consumption for catabolism + Consumption for anabolism     (4)

d [X]/dt = Consumption for active import + Synthesis in catabolism + Consumption for anabolism     (5)

#### Environment and fitness

##### 8) Environment

The environment of a cell is described by a single parameter [A_out_], the outside concentration of molecule A.

##### 9) Fitness

We assume that the fitness of an organism depends on its ability to maintain homeostasis while the environment changes. Specifically, organisms are supposed to keep [A] and [X] close to 1 mM, regardless of [A_out_]. For an organism, we calculate equilibrium concentrations [A_eq_] and [X_eq_], reached at three values of [A_out_], 0.1 mM, 1.0 mM, and 10 mM and assay deviations of the corresponding values of [A_eq_] and [X_eq_] from the desired 1 mM value. This is done as follows: if [A] ([X]) exceeds 1, then Δ[A] = [A] (Δ[X] = [X]), otherwise, Δ[A] = 1/[A] (Δ[X] = 1/[X]). For a given [A_out_], we define f_[Aout] _= 1/(Δ[A] × Δ[X]), where Δ[A] and Δ[X] correspond to [A_eq_] and [X_eq_] reached under this [A_out_]. Then, the fitness potential [18] of the organism is defined as Fp = f_0.1 _× f_1.0 _× f_10_. Obviously, Fp can vary between 0, for the least fit organisms, and 1, for the fittest organisms. Finally, F, the fitness of the organism, its expected number of offspring, can be any (non-decreasing) function of its fitness potential.

#### Numerical experiments

Typically, there are 1000 individuals in a population, and a run lasts for 5000 discrete generations. A generation consists of a selection (differential reproduction) step followed by a mutation step. Before the first generation, an initial population is created by assigning a random genotype to each individual. In the course of a selection step, organisms reproduce, and an organism produces a random number of offspring, with the expectation determined by its fitness potential. In the course of mutation, all the parameters determined by the gene, except protein type, are subject to point mutation. Major mutations can also occur.

### Some results of experiments on the evolution of the system

Computational experiments with the system just described revealed a number of behaviors, some conforming to simple biological intuitions and some others, which may appear counterintuitive. The full potential of the system obviously cannot be investigated within the framework of this initial report. Thus, here we describe only a sample of the observed patterns, which appear to be general and may have interesting biological implications.

#### General patterns

Although the system has been investigated under a wide variety of parameters and often demonstrated remarkably different dynamics, several patterns have been observed in all, or almost all, computer experiments. Below, we describe four such patterns, which at this point appear to be of primary importance.

##### 1) Dynamics of fitness potential

**Figure 2:**
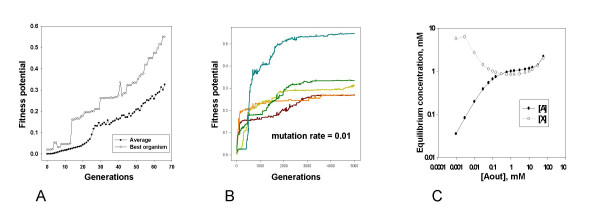
**Typical course and outcomes of evolution. Dynamics of fitness potential (A, B) and equilibrium concentrations of molecules A and X in one of the evolved organisms (C)**. **A**. Dynamics of fitness potential at the beginning of an experiment. **B. **Dynamics of fitness potential of the best organism in the population in the course of a whole experiment. Data from five independent populations are shown. **C. **The dependencies of equilibrium concentrations of molecules A and X on the concentration of A outside the cell in one of the organisms produced as the result of evolution.

Fitness potentials of randomly created organisms, used to initialize the population, are rather low (~10^-3^). Without exceptions, evolution leads to a dramatic increase of the mean fitness potential within the population, as well as of fitness potentials of the most and the least fit members of the population (Fig. 2A,B). As a rule, the growth of fitness potential involves a relatively short initial rapid phase (Fig. 2A), followed by a slow-down and by eventual plateau, usually reached after several thousands of generations (Fig. 2B).

Organisms that represent the population at the plateau phase are, in fact, rather well adapted. This is evident both from their high fitness potentials (0.25–0.60, in different runs; thus, the fitness potential in the course of a run increases by the factor of ~1000), and also from explicit consideration of their metabolism. Figure 2C shows that equilibrium values of [A] and [X] indeed remain rather close to 1 mM when [A_out_] varies between 0.1 and 10 mM, i. e. within the range which is relevant to fitness potential. Thus, the goal of selection – homeostasis of [A] and [X] – has been successfully achieved.

##### 2) Repeatability of the outcomes of evolution

**Figure 3:**
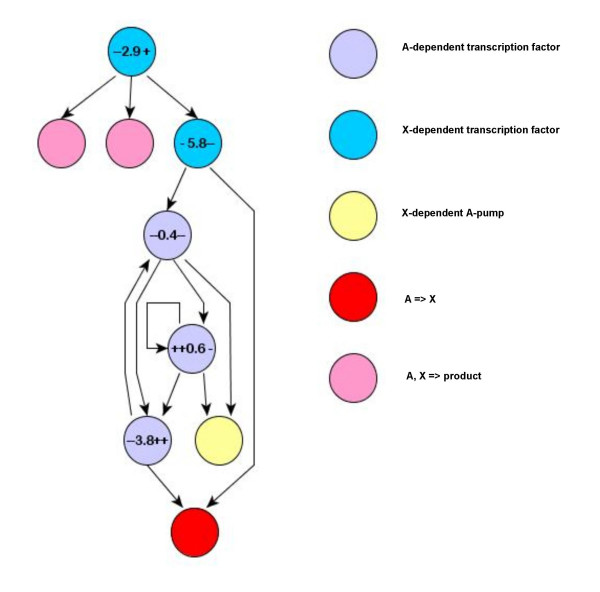
**Regulatory interactions within one of the evolved organisms**. Labels representing transcription factors are organized in the following way: numbers indicate K_d _(constant of ligand dissociation) of the TF; signs to the left of K_d _symbolize the value of EffApo (effect of a free TF on transcription), with "-" meaning 0.1 – 0.33 (strong inhibition), "- " meaning 0.33 – 0.85 (weak inhibition), "= " meaning 0.85 – 1.15 (no effect), "+ " meaning 1.15 – 3.33 (weak activation, and "++" meaning 3.33 – 10 (strong activation). Signs to the right of K_d _symbolize EffBound (the effect of the TF bound to the ligand). Fitness potential of this organism is 0.57.

When different runs are initialized with different seeds of the random number generator, they always follow different courses and the structures of evolved organisms may differ dramatically. We never observed exactly the same phenotype twice. Still, some patterns appear repeatedly and essentially every organism possesses them after long enough evolution. In particular, gene regulation through negative feedbacks is universally present in high-fitness organisms (Fig. 3).

##### 3) Impact of the population size

**Figure 4:**
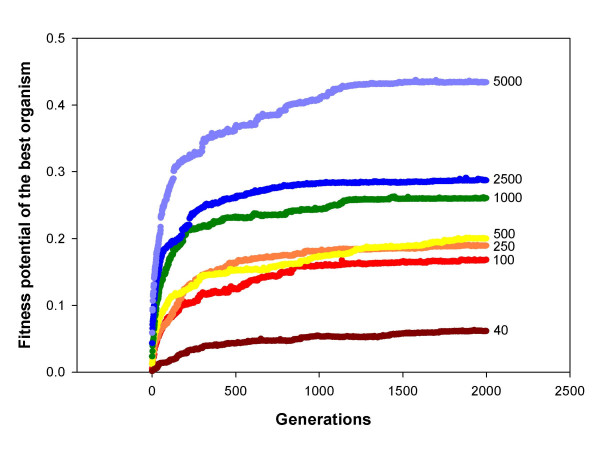
**Dynamics of the average fitness potential in populations of different size**. Each curve represents average of 10 populations. The population size is shown to the right of the corresponding curves.

When different runs were performed for populations of different sizes with all other parameters being the same, fitness potential in large populations usually grew faster and eventually reached a higher plateau. However, the impact of the population size was substantial only when it was below ~1000; after that the effect reaches saturation, *i. e*. populations of 1000 or more organisms all evolve in approximately the same way (Fig. 4). Thus, we usually studied populations of 1000 organisms.

##### 4) General properties of the evolved organisms

Organisms which emerge in our system after the mean fitness potential of the population reaches a plateau can have very different levels of complexity, despite their rather similar fitness potentials. Organisms with small genomes are intelligible throughout. Fig. 3 presents the organization of one of such simple organisms, which carries 9 genes. All the regulatory interactions in this organism are highly logical. The three A-dependent TFs are at the center form a clever regulatory switch that ensures that expression of the A-pump is enhanced at low [A] and inhibited at high [A]. Expression of the catabolism enzyme is positively controlled by the substrate (A) and negatively controlled by the product (X), as it should. Finally, expression of the two anabolism enzymes is normally inhibited but is enhanced by X when [X] becomes too high.

**Figure 5:**
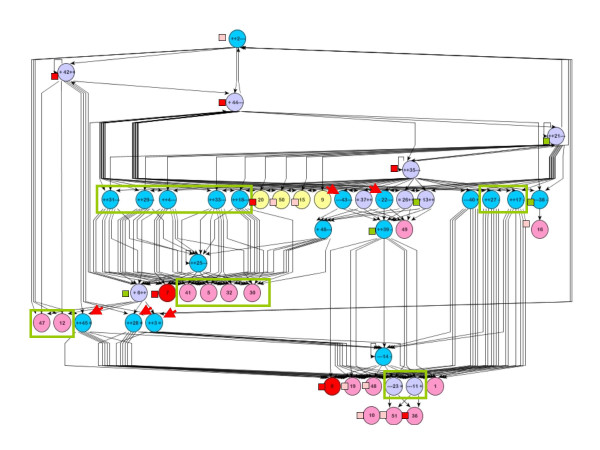
**Structure of one of complex evolved organisms**. All proteins are color-coded in the same way as in Fig. 3. Numbers on the protein labels indicate gene number in the organism.

However, not all evolved organisms are so simple and logical, and most of them contain many more genes. Fig. 5 shows the structure of one of such complex organism. It contains 51 genes (the maximum number we observed is 153 genes) and has a rather high fitness potential of 0.63, although a bird's eye view on the entire network of its regulatory interactions reveals an astounding and unintelligible complexity that is similar in many ways to the organization of many real biological regulatory networks. Still, a closer analysis shows that each particular regulatory interaction is logical: regulatory proteins create a variety of feedback loops that should stabilize [A] and [X].

**Figure 6:**
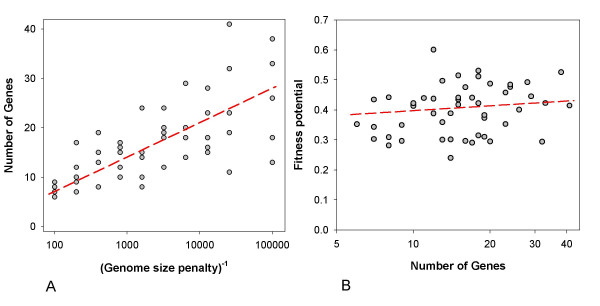
**Evolution of the genome size**. Randomly generated organisms containing ~10 genes evolved as described in the text for 5,000 generations. **A**. Dependence of the number of genes in the resulting organisms on the value of genome size penalty N (see text). **B**. Dependence of the fitness potential of evolved organisms, calculated without imposing penalty for the genome size, on the number of genes in their genomes.

The emergence of highly complex organisms would be understandable if they had higher fitness potentials than simple organisms. This, however, is not always the case. We performed multiple runs of evolution where a penalty for the number of genes was imposed. This penalty was n/N, where n is the number of genes in the organism, and N is a parameter determining severity of the penalty. The penalty was subtracted from f_0.1_, f_1.0_, and f_10_. Fig. 6A shows how the number of genes in evolved organisms is affected by N: as expected, the higher N is, the larger is the number of genes in evolved organisms.

This predictable effect was used to obtain evolved organisms with different numbers of genes. Fig. 6B shows that fitness potentials of these organisms are only mildly affected by the number of genes: after 5,000 generations, they all achieve fitness potential values within the 0.25 – 0.65 range. Indeed, selection in favor of increased complexity cannot be strong because a penalty of only ~0.001 for adding a gene is enough to prevent a substantial growth of complexity (Fig. 6A). Still, some factor encouraged the increase of complexity, which occurred even when deletions were more common than mutations which increase the number of genes (deletion bias), although mute genes which do nothing were removed from the genome under this bias, as expected. Data presented in Fig. 6 were obtained with such deletion bias.

#### Dynamics and possible irreversibility of complexity

As long as all organisms are simple at the beginning, initially their complexity (the number of genes in the genome) can only grow or, perhaps, stay roughly constant. Very complex organisms can appear only after a considerable number of generations. Still, monotonous, decelerating increase of complexity was not the only pattern we observed. Quite often, complexity rapidly increased initially, and, after reaching a temporary peak, slowly declined to a reach a lower plateau.

**Figure 7:**
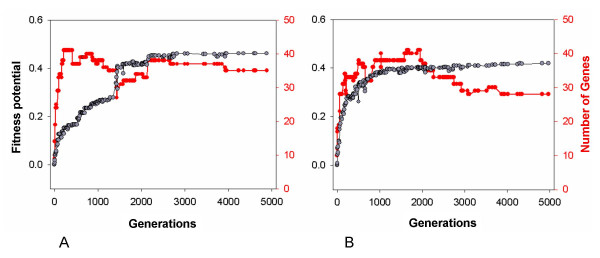
**Dynamics of genome complexity in two lineages**. Grey symbols and left vertical axes show changes in the fitness potentials of organisms in two evolving lineages. Red symbols and right vertical axes show changes in the number of genes in the organisms. The mutation rate was 0.03.

Fig. 7 shows changes in fitness potential and in the number of genes in two evolving lineages. The entire evolutionary path of a lineage in these two cases contained 200 – 250 fixations of mutations, which were more frequent at the beginning. This bias likely occurs because mutations happening in organisms that have already reached a fitness potential plateau are much more likely to reduce fitness than increase it. In contrast, there is a higher chance for a mutation to be beneficial at the beginning, and beneficial mutations become fixed. This is not to say that all mutations fixed in the course of evolution are beneficial. Grey curves in Fig. 7 show that beneficial mutations comprise a majority of fixations, but neutral and even mildly deleterious mutations can also become fixed, due to low population size.

Red curves in Fig. 7 show how genome complexity changes in two individual evolving lineages. Both lineages began their evolution from 10 genes and in both cases the ancestor organisms in the initial population contained genes encoding all five protein types. Very quickly, within the first 500 generations, the number of genes rose to 35 – 40, reaching a peak, and then remained at the same level (Fig. 7A) or declined (Fig. 7B).

Analysis of 15 similarly obtained evolutionary paths revealed that the initial rapid rise in complexity occurred in 13 cases. In 2 cases, the number of genes in an evolving lineage remained at the level of 9 – 13 throughout 5000 generations, even though these lineages ultimately achieved reasonably high fitness potentials of 0.35 – 0.55. Among 13 lineages whose complexity has risen, the number of genes reached quite diverse maximum values within the first 2000 generations, from 24 to 67. The subsequent behavior of lineages also varied: some kept nearly the same complexities that were attained during their initial rises, while others became considerably simpler. In three cases, the number of genes ultimately fell to as few as 8 – 13.

#### Origin of non-essential genes

Different genes make very different contributions to fitness potentials of organisms which evolve in our simulations. The contribution of a gene to the fitness potential can be defined as its relative change when the gene is deleted. Let us call genes whose deletion reduce fitness potential by more than 3% essential, and the remaining genes nonessential. We investigated contributions of genes to fitness potentials in a number of evolved organisms, with highly consistent results.

In simple organisms (almost) every gene is essential. However, in complex organisms like the one shown in Fig. 5 the story is entirely different. Only a small number of genes, indicated by red squares to the left of them, are critical: deletion of any of these genes reduces fitness potential by at least 80%. A few other genes, marked by pink squares, are significantly less important: their deletion reduces fitness potential by 3 – 15%. The majority of genes, however, are nearly dispensable: their deletion reduces fitness by less than 3%, and in most cases by less than 0.5%. There are even some genes, marked by green squares, whose deletion increases fitness potential by up to 0.5%.

**Figure 8:**
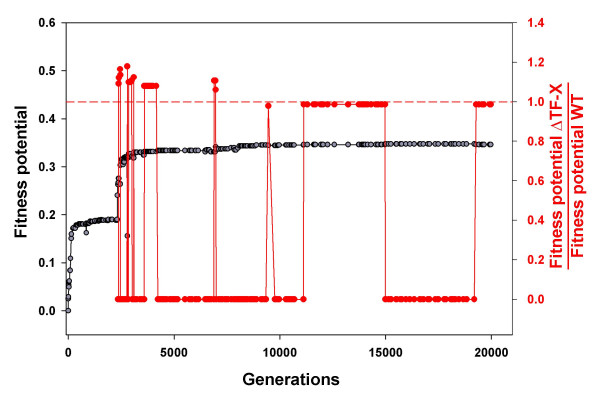
**Dynamics of essentiality of an X-responsive transcription factor gene in an evolving lineage**. While fitness potential (grey line) increases, the impact of knocking-out of the gene (red line) fluctuates. Essentiality of the gene keeps changing even when fitness potential reached the plateau.

Fig. 8 shows the data on a lineage which evolved for 20,000 generations. We consider one gene, which encodes an X-responsive TF. This TF inhibits expression of two catabolism enzymes upon binding X, a reasonable function which stabilizes [X]. However, this TF is nonessential: after it emerged in generation 2344, fitness potential of artificially generated deletion mutants comprises 98.7% of the fitness potential of wild type organisms. Red symbols in Fig. 8 show how the contribution of this gene to fitness potential evolved with time. The essentiality of this gene changed repeatedly. At different times, the gene was either critical (its deletion reduced fitness potential more than 10-fold), slightly beneficial, or even detrimental (its deletion increased fitness potential slightly). Similar frequent switches of essentiality were detected in all the genes that were studied in detail, including those encoding TFs, enzymes, and A-pump.

## Discussion

We pursued an ambitious goal: to use a variant of a genetic algorithm to evolve a virtual but biologically relevant function, with components that mimic real biological entities (promoters, operators, enzymes, membrane transporters, transcriptional regulators) and follow the basic laws of biochemistry. Our attempt was crowned with some success: not only did our virtual organisms develop the desired function, but the ways in which this was accomplished were remarkably similar to what apparently happens in nature. Evolved organisms established appropriate combinations of negative and positive feedback loops, which sometimes had rather elegant designs but more frequently appeared to be excessively complex. Maybe most strikingly, a large number of nonessential genes were often present. Whereas the excessive complexity and the presence of nonessential genes in living organisms can be explained by invoking multiple unknown selective factors, with virtual organisms this explanation is impossible, since we imposed the selection criteria ourselves. Our results suggest that many peculiar and sometimes counterintuitive properties of real biological systems may represent as yet unexplored by-products of the Darwinian evolution and do not necessarily implicate unknown biological mechanisms or selective factors. Let us review our findings systematically.

### General patterns

The maximum value of the fitness potential we have ever observed for a random organism is 0.07, but usually it is below 10^-4^. This means that it is essentially impossible to stochastically generate an organism capable of keeping concentrations of A and X close to 1.0 mM. In other words, we were asking the organisms to evolve a rather difficult function, and initializing the population with random organisms leaves ample room for improvement.

Overall dynamics of fitness potential is highly consistent in different experiments and is what one would expect: an initially rapid increase is followed by a slow-down, and eventually a plateau is reached (Fig. 2A,B). In fact, since the slow-down is slow itself, asymptotic behavior of fitness potential is hard to investigate, and we cannot rule out its marginal growth even after ~5000 generations. However, it seems certain that most of the increase of fitness potential occurs in the first several thousands of generations, a dynamics expected if the population climbs a smooth fitness peak.

Obviously, the number of fitness potential peaks in the space of all possible genotypes of our organisms must be huge, and chance plays a significant role in which trajectory an evolving population will choose. Indeed, independent runs performed under the same parameters never yielded identical outcomes, so that, at the very least, the number of peaks greatly exceeds the number of experiments performed so far. The existence of multiple solutions for the same complex problem appears to be a ubiquitous phenomenon (e. g., [19]), and very different alternative solutions are often of similar quality. For example, many substantially different neural networks can recognize a pattern with approximately the same success [20]. In our case, different solutions of the problem of maintaining homeostasis often involve very different levels of complexity.

Adaptive evolution in our experiments was always facilitated when the size of the evolving population increased. One can expect that a higher population size should increase the rate of adaptation, since positive selection is more efficient in large populations. However, it seems that not only the rate of the growth of fitness, but even the height of the plateau, to be reached eventually, increased with the population size (Fig. 4). This second observation, if correct, may be explained in at least three ways. First, a large population explores a larger space of initial conditions and, thus, can reach a higher peak. Second, more efficient selection in a large population can prevent slightly deleterious mutations from being irreversibly incorporated into the design of organisms, which could trap them on relatively lower fitness peaks. Third, more efficient selection in a large population means that, among several beneficial mutations, the best one will be fixed with high probability, making sure that the optimal design and the highest fitness peak will be reached. Additional experiments are necessary to figure out which of these, not mutually exclusive, mechanisms are responsible for higher asymptotic fitness values reached by larger populations. However, increasing the population size past several thousands of individuals did not result in further increase of the asymptotic fitness.

### Dynamics of complexity: initial increase with subsequent simplification

There is an ingrained belief among experimental biologists that living beings are structured in an orderly way and that the goal of biology is to uncover this order. This belief does not come from nowhere: many well-studied systems are indeed highly ordered and efficient, to the point that they even look like a product of engineering design. In every area of biology we see multiple examples of hierarchical modular organization that resembles the design of man-made machines and mechanisms. In such systems it is usually easy to assign specific functions to particular genes, proteins, protein complexes, cells, tissues, and organs.

However, there is another side to this coin. Even though many biological systems appear to have rational designs, others do not. For example, the majority of regulatory pathways contain a multitude of interacting components (the system of blood clotting, the system of complement, the system of regulation of cell proliferation in multicellular organisms, to name just a few). Each particular interaction in such systems makes sense, but it appears that a much smaller and simpler overall design could have sufficed. This apparently excessive complexity is further confounded by the relatively recent realization that close to 80% of genes in the genomes are nonessential, in the sense that they can be individually knocked-out with only limited effect on the organism fitness [21-26].

Two explanations of such excessive complexity are usually invoked. According to the first of them, evolution is a series of "frozen accidents" and excessive complexity is a result of a pileup of adaptive mechanisms, with more recent mechanisms using elements of the older ones [27]. The alternative explanation, preferred by many experimental biologists, if sometimes only subconsciously, is that we cannot reproduce in the laboratory all the details of the environment in which an organism lives in nature. Therefore, the seemingly nonessential genes and the apparently overly complex organization of many biological systems represent an adaptation to the complex combination of largely unknown environmental factors.

This alternative explanation is a substantial departure from the widely accepted paradigm of biology, according to which there must be a biological necessity behind every aspect of any living system. If, as our results appear to imply, excessive complexity and nonessential genes can arise due to circumstantial reasons and not in response to direct evolutionary pressure, this may make biologists suspect similar reasons behind the existence of numerous control circuits and redundant genes in living organisms. These considerations prompted us to focus on elucidation of the origins of excessive complexity and of nonessential genes.

So, what drives the almost universally occurring increase in the number of genes during evolution of our virtual organisms? This increase occurs even when deletion mutations in our system were more frequent than duplications, genetic exchange and random gene insertions put together. It is not clear what evolutionary benefit organisms achieve by becoming more complex since fitness of evolved organisms does not seem to depend strongly on the number of genes (Fig. 6B). Our results suggest that in the argument between "adaptationists" and the adherents of the theory of "frozen accidents" the latter are much closer to the truth. Indeed, complex organisms arise in our model without any overriding need for complexity since the selective pressure that is applied to evolving organisms is simple and well-defined. Furthermore, organisms sometimes achieve good adaptation to this pressure in a simple way, without accompanying complexity.

Still, the frozen accidents explanation does not seem to describe fully the dynamics of complexity in our system. Indeed, according to this explanation the rise of complexity should accompany the rise of fitness, because new elements are added on top of the old ones in order to improve the level of adaptation. This, however, does not seem to be always the case in our model. Whereas in Fig. 7B the rise of complexity is concomitant with the rise of fitness potential, in Fig. 7A they are correlated very poorly. The very abrupt complexity burst in this case corresponds to a relatively modest rise of fitness potential, while subsequent, more pronounced rise of fitness potential happens when complexity is actually somewhat declining. Similarly poor correlations were observed in many other evolutionary paths that we examined, suggesting that the rise of complexity is a process that does not necessarily parallel adaptation and seems to abide its own laws.

Thus, our work may suggest that other mechanisms, different from both adaptation to complex environments and from accumulation of frozen accidents, also play a role in the emergence of complex systems. Let us consider four hypotheses on what these mechanisms might be. The first two of them envision direct gain of fitness as a result of an increase in genome size. The remaining two hypotheses envision increased evolvability due to an increase in genome size.

#### 1) Multiple peaks and ridges

The best way to describe this hypothesis is to consider evolutionary process as climbing of peaks on the fitness landscape. If we imagine that organisms with small and large genomes climb the same mountain by hopping from peak to peak or by following ridges on the fitness landscape, then at the end all of them will both reach the same highest peak and achieve the same level of fitness, as it appears to be the case (Fig. 6B). However, if small-genome organisms have only a few peaks to hop on and only a few ridges to follow, while large-genome organisms have an opportunity to ascend along multiple peaks and ridges, then in the process of climbing large-genome organisms will have an advantage, due to a better chance to stumble upon a peak or a ridge. In other words, we assume that a fraction of beneficial mutations is higher among mutations that increase the genome size than among mutations that decrease or preserve it. If genome-increasing mutations create more organisms with improved fitness, there is a better chance for them to reach higher fitness values. As a result, evolution will lead to increased complexity.

This hypothesis rests on an assumption that fitness landscapes of organisms with larger genomes have a higher density of peaks and ridges. This assumption can be tested directly. Suppose that we create a large number of random organisms of different genome sizes, *e. g*., with 10 genes and 20 genes, and evaluate their fitness values, thus obtaining sets F^10 ^and F^20^. If the assumption is correct, then F^20 ^should contain more high points than F^10^. This does not necessarily mean that the average of F^20 ^must be higher than of F^10^. We expect, however, the distribution of fitness in F^20 ^to have a significantly longer right-side tail than in F^10^. This test is worth being performed.

#### 2) Missing schema element

This hypothesis assumes that at the beginning of evolution organisms have a number of potential mechanisms that could help them to achieve homeostasis of metabolites but these mechanisms are not functioning or functioning poorly because the genome is missing a gene encoding an important component of the mechanism (*e.g*., a transcriptional regulator, an enzyme, or a pump with certain properties). In the language of evolutionary computation theory, these organisms have potentially beneficial schemata (see ref. 3, 4, 28) which are missing one element. The more genes an organism acquires as a result of mutation, the higher the chances that this missing element will be present among newly acquired genes and the homeostasis mechanism will start working. Mutations which do not change or even reduce the number of genes do not bring new elements to the system and the evolution can proceed only through a more complicated path of circumventing the absence of a critical element by adjusting parameters of the presumptive homeostasis mechanism, making it to work without it. One advantage of this hypothesis is that it readily explains the existence of lineages that do not undergo a complexity burst: they may initially lack the postulated nearly complete potential homeostasis mechanisms.

If this hypothesis is correct, than 1) randomly created organisms with large genomes should have not-too-small fitness values much more often than organisms with small genomes, because there is a higher chance for them to have a completely formed homeostasis mechanism and 2) large-genome organisms with low fitness values should have more presumptive mechanisms of homeostasis than small-genome organisms. Indeed, the number of possible schemata in a genome with r genes is proportional to a^r^, where a is the number of states in which a gene can exist [10,11]. The same obviously applies to incomplete schemata. Prediction 1 can be directly tested in the experiment proposed for the hypothesis of "multiple peaks and ridges". Prediction 2 will allow us to discriminate between the two hypotheses and can be tested in the following experiment.

Let us create two populations of random organisms with 10 and 20 genes and choose pairs of organisms with different number of genes but similar levels of fitness within a pair. Then, each organism will be used to create a population of 10,000 of its copies and each population will be subject to random gene insertions so that each organism receives one extra gene. If the hypothesis of "missing schema element" is correct, we should expect a larger fraction of beneficial mutations in organisms with 20 genes than in organisms with 10 genes, since large-genome organisms should have more incomplete schemata that an additional gene can complete. This can be evaluated by assessing the presence of a prominent right shoulder in the distributions of fitness after insertions.

#### 3) Higher mutation rate

This is probably the most straightforward hypothesis of all and the one which is the easiest to test. It postulates that since the rates of many mutations, namely duplications, deletions, and point and operator mutations, were defined per gene, an increase in the number of genes leads to effective increase of the per genome mutation rate. This gives an advantage to large-genome organisms, since they make more attempts at gaining in fitness. Consequently, larger-genome organisms advance faster which leads to increased average complexity. This hypothesis can be tested by introducing mutations on per genome basis. If this hypothesis is correct, we expect complete disappearance of complexity bursts.

#### 4) Better evolutionary memory

Evolving organisms need to be able to restructure their genome without losing too much of the accumulated fitness along the way. In other words, peaks and ridges on the fitness landscape should not be separated by valleys that are too deep. If this is the case, an evolving organism could move from one peak or ridge to another with greater ease, since stepping into a fitness valley will not necessarily eliminate it from the population. This property can be formulated as "evolutionary memory" – how well do organisms remember past evolutionary achievements when they undergo a mutation. It seems natural to assume that large-genome organisms should have better evolutionary memory (more shallow fitness valleys) than small-genome organisms, because the responsibility for fitness is spread among more genes. Thus, a mutation is less likely to have a devastating effect.

This hypothesis predicts that fitness of larger organisms should be more resistant to the destructive effects of mutations. This conclusion can be tested by creating populations from copies of moderately fit organisms (F ~0.1 – 0.2) and mutagenize them with mutations of different types thus creating populations G^r+ ^_i_, G^r- ^_i _and G^r ^_i_. These populations will be subjected to mutagenesis with a combination of mutations used in our model at their standard ratio. If the hypothesis is correct, an average fitness of mutagenized population normalized by the average fitness of the original population should be higher for organisms with larger genomes.

#### 5) Discriminating between the explanations for increased complexity

Above, each kind of hypotheses that explain the increase in complexity was represented by only two examples, whereas other hypotheses are also feasible. For example, the same considerations that led us to introduce the hypothesis of evolutionary memory allow one to hypothesize that larger organisms have smoother fitness landscapes. Consequently, these organisms would be able to move up the gradient of fitness in longer stretches that are not interrupted by valleys and ravines. Also, the increased complexity can be a purely selectively neutral phenomenon: mutations that increase complexity have a higher chance of being effectively neutral, while mutations that reduce it are more likely to be deleterious. Further experiments will be necessary to test all these hypotheses. Indeed, it is quite possible that the burst of complexity has many driving forces.

In addition, some lineages do not experience increased complexity in the course of their evolution. It may be interesting to find the reasons for this difference in behaviour by identifying structural similarities between organisms whose evolution does not involve complexity burst. This, in itself, may be sufficient to identify the mechanisms underlying the increase in complexity.

#### 6) Causes of secondary simplification of organisms

Our results show that complexity of fully evolved organisms is determined by a combination of at least two evolutionary trends (Fig. 7). First, there is a tendency of organisms to become much more complex at the early stages of evolution, and, second, there is a tendency of complex organisms to become simpler at the later stages of evolution. Neither of these trends is absolute: there are lineages that do not become more complex at the beginning, just like there are lineages that do not become simpler at the end. Interestingly, organisms that possess elegant and economical organization of their gene network (like the one in Fig. 3) appear to arise in two different ways: either by remaining simple throughout their evolution and developing homeostasis of [A] and [X] through adjustment of kinetic constants alone, or by first becoming highly complex and then going through the process of simplification.

What are the reasons for the reversal of the direction in which complexity changes? Perhaps, in large-genome organisms some force directed towards reduction of the genome size becomes stronger. At some point, when the drive to increase complexity declines due to diminishing returns and the drive to reduce complexity increases due to the large size of genome, the two competing forces become equal and this stabilizes genome size. Later, with the decline of the force that makes genomes grow, the competing force causes their simplification.

What can be the nature of the force directed towards simplification? One obvious candidate is the penalty imposed on the fitness value of an organism for the large genome size. However, although the size of the genome is certainly affected by the penalty if it is high enough (Fig. 6A), it reaches a plateau and then declines even if this penalty is very small or nonexistent (not shown). The second possibility is the mutation load. Our preliminary results show that with an increase of mutation rate to very high levels (0.3 – 1.0 instead of 0.03 that we used in experiments described here), the complexity of evolved organisms becomes noticeably smaller (data not reported). Then, small-genome organisms survive the destructive effect of mutations on their fitness better than large-genome ones, due to lower per genome mutation rates.

However, we prefer a simpler explanation of the changing genome complexity in the course of evolution. There may be no special force that reduces genome size other than the fact that, in the corresponding experiments, genome-decreasing mutations were 10% more frequent than genome-increasing mutations. The plateau and the following slow decline in complexity may ensue simply because the driving force of the complexity burst, whatever it could be, disappears after a few hundred generations. Obviously, the way to test this hypothesis is to see whether reduction of complexity will be observed in the absence of this mutational bias.

It seems that functional biological systems sometimes appear through simplification of systems that experienced a temporal peak of complexity. Indeed, there may be no direct path from something simple to something complex, because the only evolutionarily feasible paths, alone which fitness always goes up, go through a supercomplex phase. Apparently, many overcomplicated pathways are evolutionarily young (TNF, complement, coagulation, etc.). If mature adaptations are secondarily streamlined, this may contribute to irreversibility of their evolution.

### Origin of non-essential genes

Our model organisms are prone to acquire large numbers of non-essential genes, despite a rather simple criterion that we used for selection. One could imagine that even in a very complex genome all or almost all genes are, nevertheless, essential. However, this is not the case, and a large fraction of genes is only marginally relevant to fitness. Similar results were obtained in real biological experiments that analyzed how gene knock-outs in yeast affect the fitness [23]. In competition with the wild type, ~85% of knock-outs led to less than 20% reduction of fitness and a few knock outs even slightly increased fitness.

A trivial explanation for the existence of multiple nonessential genes is that deletions are not frequent enough to eliminate them. However, evolved organisms only rarely contain truly mute genes, such as genes encoding TFs not interacting with any operators. On the contrary, practically all nonessential TFs seem to be fully involved in the organism biochemistry and create entirely reasonable negative and positive feedback loops that should lead to stabilization of [A] and [X]. The organism shown in Fig. 5 is replete with regulators that control gene expression in a seemingly beneficial manner, yet they can be deleted with only minor effect on fitness. This result raises three questions: what factors lead to the persistence of these nonessential genes in the genome; if these genes are nonessential and are not subject to selection, why and how do they develop proper functions; and, conversely, if they perform all the proper functions, why are they nonessential?

Fig. 8 shows that genes that are nonessential in evolved organisms could have spent a significant amount of time during organism evolution being essential and protected from deletions. This explains why a non-essential gene may seem to perform a proper function and, nevertheless, be nearly neutral. Apparently, the structure of organisms frequently shifts during evolution, making some processes more important and some less important at different moments. This strongly influences relative effects of different genes on fitness.

Thus, only a fraction of genes of the genome contributes to fitness of any given organism. This is a rather novel view on cell biochemistry. It is worthwhile to analyze how the levels of essentiality of all the genes of an organism are distributed. Do they tend to alternate between the extremes, 0 and 1, as it appears from Fig. 8, or they can also have intermediate values? As much as the preliminary analysis of four organisms can be an indication, it appears that the situation differs in different organisms. In some organisms, most of genes do indeed have essentiality values close to either 0 or 1, while in others a majority of genes have intermediate values of essentiality. It is conceivable that at the beginning of evolution an organism makes a choice between "collective biochemistry" where every gene contributes somewhat to fitness, and "alternate biochemistry" where fitness is determined by one of several alternating pathways, and then sticks to this choice throughout its evolution. It will be also interesting to see if the type of biochemistry correlates with the presence or absence of complexity burst and with the extent of simplification that organisms experience during evolution.

The peculiar alternating behavior of essentiality of a gene can be regarded as merely a fact of no particular importance other than as an explanation of the longevity of genes that appear nonessential. Alternatively, it can be seen as an important factor of evolution, which, for example, provides a backup metabolism on which an organism can rely if the pathway that is currently important is damaged by mutation. There are also several other considerations that suggest that having an extra set of functional genes can be of help for successful evolution. It will be interesting to determine if preservation of nonessential genes facilitates evolution.

Our data indicate that simple Darwinian selection somehow leads to the accumulation of genes with seemingly logical but unimportant functions. Searching for functions of uncharacterized genes, either discovered in sequenced genomes or identified as genes encoding "interactors" of known proteins, has become one of the main activities of experimental biologists. Our results suggest, however, that such a search can conceivably be futile in many cases. Nonessential genes can be present in the genomes for no other reason than the quirky behavior of the evolutionary process. In fact, experimental studies can be even deceiving by suggesting an artifactual assignment of a gene function. We found that in some cases deletion of a gene in an evolved virtual organism does not change the fitness but affects biochemistry of the organism at extreme conditions. For example, deletion of some genes significantly reduces [A] at [A_out_] = 0.01 mM, but has little effect with [A_out_] within the 0.1 – 10 mM range, for which the organisms were selected. If such an observation was made in a real biological experiment, an investigator would conclude that the role of the deleted gene is to maintain [A] at a very low [A_out_]. We know, however, that our organisms have never encountered such a low [A_out_], and were never selected for this function. Therefore, this assignment of a function would be entirely incorrect. It is possible that many functions currently attributed to specific genes in real organisms on the basis of mutational analysis have a similar artifactual nature.

A somewhat less trivial explanation for a high proportion of non-essential genes is that they may be present in multiple copies, and therefore deletion of only one of these copies causes very small effect. Indeed, evolved organisms contain numerous genes that are exact or nearly exact copies of each other, thus forming identical regulatory connections. We analyzed several such groups of paralogs, with rather diverse results. In some cases (Fig. 5, marked by red arrows) deletion of all sister genes dramatically reduces fitness, clearly supporting this explanation. In the majority of cases, however, the deletion of an entire group of sister genes (enclosed by green squares) has only a small effect on fitness (less than 0.5%). Conditional non-essentiality of a gene, such that a non-essential gene becomes essential after another gene is knocked-out, was very rare in our experiments (data not reported).

## Conclusion

We described a system that makes it possible to model evolution of primitive but functional cells. Although intentionally simple, this system involves all levels of organization inside the cell, and computer experiments demonstrates a remarkably rich variety of evolutionary dynamics. Some of the phenomena we observed were expected, while others, such as the tendency of many evolving organisms to become very complex and to accumulate a large number of non-essential genes, did not yet receive a simple, certain explanation.

The potential applications of our system are not limited to addressing the issues of complexity and gene redundancy, and may include many other salient properties of living systems, such as emergence of modularity and robustness. The fact that we simulate evolution of life-like virtual organisms rather than of purely abstract computer creations is, in our opinion, very important. First, the model takes into account unique properties of biological systems. Second, the solutions provided by the model are immediately expressed in the language of biological concepts (genomes, genes, gene expression and its regulation, proteins, fluxes of metabolites, etc.) rather than mathematical abstractions and as such have infinitely better chance to affect the thinking of biologists.

## Methods

### Computational details

#### 1) Initialization

For each run, an initial population is created as follows. Each organism has 10 ± √10 genes (with Gaussian distribution). Each gene is chosen randomly, with equal probability, to encode a pump, an enzyme, or a TF. Again at random, half of the enzymes are catabolism enzymes and the rest are anabolism enzymes. Similarly, half of TFs are A-dependent and the rest are X-dependent. Each gene is assigned at random one of ten types of operators, and each TF is assigned at random one of ten types of operators to which it binds. Finally, all quantitative constants: Pst, K_MA_, K_MX_, V_max _for pumps and enzymes, and Pst, K_d_, K_b_, EffApo and EffBound for TFs, are assigned at random a value between 0.1 and 10, with the center of distribution at 1.0. This latter operation was performed by assigning to each constant a value of 10^a^, with a being a uniformly distributed random number between -1 and +1.

#### 2) Selection

For each of the 3 outside concentrations of A ([A_out_] = 0.1, 1.0, or 10.0), we determined equilibrium values of [A] and [X] by integrating differential equations (4) and (5) numerically, using the standard Runge-Kutta algorithm. We assumed that an equilibrium is reached when the concentrations of proteins and of molecules A and X at the subsequent time steps changed by less than 0.1%.

Different equilibria can be reached from different initial concentrations, if the equations possess multiple stable equilibria. We did not investigate this issue in detail and mostly used just one set of initial concentrations: 0.5 mmol for [A] and [X] and 1 mmol for each gene product. In fact, some observations suggest that equations (4) and (5) often have only one stable equilibrium. Non-equilibrium stable attractors are also possible. Indeed, in ~25% of cases, the concentrations oscillated indefinitely, and the integrator did not converge to equilibrium after 5000 iterations. In such cases, an organism was assigned a zero fitness potential.

After fitness potentials of all the organisms in the population have been evaluated, their differential reproduction occurs. We tested several ways for this process and all of them produced similar results. In most of the experiments presented in this paper, the probability of an old organism being copied into the new generation is proportional to the value of 2^Fp^-1, where Fp is its fitness potential. Thus, organisms with Fp = 0 do not reproduce, and fitness is a rapidly increasing function of fitness potential. Copying continues until a new population of 1000 organisms is recruited, which ensures that the population size remains constant, without affecting relative fitness values of the genotypes.

#### 3) Mutation

Point mutations change constants encoded in genes. A point mutation affecting a continuously-varying constant is produced by multiplying the existing constants by 10^a^, where a is a uniformly distributed random number bound between -0.5 and +0.5. Thus, the value of these constants can either increase or decrease but not more than by a factor of ~3.33. If, after a mutation, the value of a constant falls below 0.1 or rises above 10, it is made equal to 0.1 or 10, respectively. A point mutation affecting either the type of an operator or the type of a TF is produced by randomly choosing the new value of the type (between 1 and 10), regardless of its current value. In some experiments, deterioration bias is introduced into point mutation, so that V_max _and Pst decrease more often than increase and K_m _more often increase than decrease.

Major mutations occur by randomly choosing a string of 1 – 5 genes within the genome and performing one of three operations: transfer, where the string is copied into a random position within the chromosome of a randomly chosen organism of the population; duplication, where a string is copied into a random position within the chromosome of the same organism; and deletion, where the string is deleted from the chromosome. We also considered insertions of a random gene, which is inserted into a random position within the genome. This latter mutation imitates either horizontal gene transfer or a situation when some other protein of a given organism starts to interact with A or X and thus becomes involved in the function that is undergoing evolution. The combined frequency of mutations adding genes to the population (gene transfer, duplication and insertion) was approximately 10% lower than the frequency of deletions, so that mutation on average results in slightly less complex organisms.

The frequencies of all kinds of mutations are determined by the global parameter Mutation Rate, with the rates of different types of mutations being multiples of this parameter. The coefficients of these multiples are chosen in such a way that at Mutation Rate = 1.0 in one cycle of mutagenesis each gene undergoes on average 0.1 major mutations and 0.1 point mutation. A higher or lower Mutation Rate proportionally increases or decreases this number.

#### 4) Some specific assumptions

In all our experiments several parameters did not change. Catabolism always involved production of 4 X molecules from one A molecule. This value was chosen as a compromise between the efficiency of glycolysis (2 molecules of ATP/molecule of glucose) and oxidative phosphorylation (36 molecules of ATP/molecule of glucose). Anabolism always involved consumption of equal numbers of A and X molecules, and one molecule of X was always required to import one molecule of A. Each TF could bind to operators of only one (matching) of the 10 possible types. We set Perm = 0.1 and Degr = 1.0.

### Implementation of the software tool

The main body of the program has been implemented with Python programming language [29] using version 2.4 by ActiveState [30]. The most computationally intensive piece of code, namely the fifth-order Cash-Karp Runge-Kutta method for integration of ordinary differential equations, was implemented in C++ after [31] as a separate dynamic link library (dll) built using Microsoft Visual C++ 6. A Python-compatibility layer was generated using SWIG tool [32]. The software tool is available on request.

## Authors' contributions

AAN conceived and piloted the project, suggested the model, participated in the software implementation, conducted and interpreted the experiments. NNB conducted and interpreted the experiments, participated in the software implementation. LJM designed and implemented the software, suggested experiments, participated in analysis and interpretation of the results. AAN wrote the initial draft of the manuscript but has not lived to see it in its final form. NNB and LJM produced the final version of the manuscript.

## Reviewers' comments

*Authors' response:*

The authors wish to thank the three reviewers for their thoughtful suggestions. A number of these suggestions have been taken into account. However, because the first author could not participate in the process, we wanted to be conservative with our revisions.

*Reviewer's report 1*

Dr. Eugene V. Koonin, National Center for Biotechnology Information, NIH, Bethesda, MD

This is a decidedly fascinating paper, even if the results and conclusions, in my opinion, should be viewed as preliminary. The object of study is "virtual life" but, unlike many other studies in this area, where the evolving entities are abstract algorithms or strings of symbols, the model here, although very simple, is rendered in biological terms. The richness of the model, which includes of just 5 virtual proteins, is astonishing – a huge variety of evolutionary trajectories. Probably, the single most interesting results is the frequent, rapid emergence of excessive complexity, with subsequent simplification as the systems is optimized by selection. It does not seem to be the case, however, that this or any other particular regime dominates the evolution of this system, rather, it seems that the evolutionary trajectories are chosen purely stochastically. Should we make a leap of faith (admittedly, it is a considerable one) and accept that the evolutionary modalities of this simple, virtual system reflects those of real biological systems, this gives us a genuinely novel outlook at the evolution of complexity. As I understand this new view, evolution of complexity (and, more specifically, modularity, redundancy, robustness etc etc) is neither an inevitable adaptation nor a "syndrome" caused by relaxed selection (as in the popular hypothesis of Lynch and Conery) but rather just one of the modes of evolution that is randomly, i.e., supposedly, due to imperceptible fluctuations (as far as we can say), chosen by some lineages but not others.

I think it is too early to subscribe to this new concept of biological complexity (in a sense, a new vision of biological evolution in general) but what seems to be beyond doubt and is, probably, the main result of this work is that the model described here has a vast potential for further development and analysis. Thus, the present work is likely to spawn a novel, major research direction and, in that sense at least, is a striking success.

Aside from these general considerations, here are several questions and comments that the authors might consider when revising the manuscript.

1. It is hard for me to fully understand the logic of the discourse in the "**Dynamics and possible irreversibility of complexity" **section of the Discussion. The authors consider two alternative explanations of the "excessive" complexity of some biological systems:

i) the contingent nature of evolution

ii) hidden, conditional essentiality of many genes

They then claim that the second explanation contradicts "naïve pan-adaptationism" (my wording but I think this is the gist of what is said). Either I misunderstand something or it is the other way around, i.e., the first explanation is, more or less, non-adaptationist, and this is what follows from the next paragraph. I believe this deserves attention.

2. Also, with regard to the same issue of the role of contingency in evolution, I think it would make a lot of sense to cite not only Gould-Lewontin but also equally (if not more) relevant papers by Francois Jacob on "evolution and tinkering" (Science. 1977 Jun 10;196(4295):1161-6; Ann N Y Acad Sci. 2001 Apr;929:71-3)

3. In the discussion of the possible causes of the decrease in complexity, we read:

"However, although the size of the genome is certainly affected by the penalty if it is high enough (Fig. 6A), it reaches a plateau and then declines even if this penalty is very small or nonexistent (not shown)." This is a really important issue, and the way it is treated leaves some dissatisfaction – why not showing these results as long as they have been obtained? I think the paper would substantially gain if the authors made an effort to be more specific about the effect of genome-size penalty (this is one of the main problems that make me believe that these results should be considered preliminary).

*Reviewer's report 2*

Dr. Shamil Sunyaev, Harvard Medical School, Boston, MA

This very interesting manuscript presents a toy computational model of an evolving cell. Although the model has a high level of abstraction, it incorporates many features of a real living cell. Several types of mutational events are allowed. Fitness of an organism is somewhat arbitrary, although reasonably, defined as the ability to maintain homeostasis.

The model allows investigating the gene network structure of organisms reaching high fitness. These structures appear to be surprisingly diverse given relative simplicity of the model. The authors specifically focus on number of genes, which they consider a proxy to "complexity". This analysis provided a few interesting counterintuitive results.

The discussion section of the manuscript is interesting but sometimes speculative. It probably can be made clearer and concise. Some explanations of the "complexity burst" can be possibly merged. Also, high per genome mutation rate is proposed to explain both increase and reduction of complexity.

In sum, the manuscript convincingly shows that simple computational models can contribute greatly to current studies on evolution of biochemical and regulatory networks.

*Reviewer's report 3*

Dr. Arcady Mushegian, Stowers Institute for Medical Research and University of Kansas Medical Center, Kansas City, MO

### Abstract

#### Background

None of the properties discussed here (optimality, complexity, modularity, robustness, and evolvability) are exclusively organism-level; cf Andreas Wagner's book (Robustness and Evolvability in Living Systems; Princeton University Press, 2005)

I do not think these paragraphs are germane to the paper. Indeed, consider condensing them into one sentence:

"Computer scientists, impressed by the beauty and apparent universality of Darwin's theory, proposed a concept of genetic algorithm, a software environment in which strings of information ("chromosomes") undergo repetitive cycles of fitness determination, selection, multiplication, and mutation/recombination [2,3]. Gradually, the strings evolve and become increasingly fit to the goal set by the experimenter. Genetic algorithms proved to be efficient in diverse optimization tasks in engineering [4,5] and software design [6].

This methodology, together with some other achievements such as theory of automata, led to creation of what became known as "artificial life" (see ref. 7 for review). Virtual organisms which constitute artificial life ("Life", "Polyworld", "Tierra", "Avida", etc.) consist of pieces of program code that either compete for computer memory or try to outperform each other in logical operations or mathematical calculations. Thus, definitions of fitness in artificial life experiments are usually biologically meaningless."

"Such modeling studies are instructive from a biochemical perspective and will likely have a strong impact on biotechnology, but they tell us little about evolution of life *per se*." – What does this mean – evolution as it actually took place to produce the observed life forms? Or behavior of other models, such as yours?

#### Results

##### Some results of experiments on the evolution of the system

General patterns

**2) Repeatability of the outcomes of evolution**

The reported results require some statistics: if, say, all variables describing phenotypes are independent, how non-trivial is the observation that phenotypes were not observed twice? On the other hand, when 'some patterns appear repeatedly', how does that compare with random generation of phenotypes?

**3) Impact of the population size**

These observations should be related to Michael Lynch's work (pubmed 14631042 and 16280547) In fact, much of the following is best discussed in light of Lynch/Conery theory.

##### Origin of non-essential genes

An optional but perhaps an interesting question, in line with A. Wagner's thinking about fitness: if some of these non-essential genes are knocked out, are contributions of the remaining genes to fitness affected (sudden appearance of new essential genes maybe?)

#### Discussion

Did authors check distribution of different types of loops/motifs viz. what is observed in real cells (cf. Uri Alon's work on network motif frequencies in E.coli network)? Authors may want to indicate that this should be done at least.

General patterns

"Adaptive evolution in our experiments was always facilitated when the size of the evolving population increased "-above 1000; but the Lynch zone may be important, see above.

##### Irreversible evolution of complexity

**4) Better evolutionary memory**

Role of duplications (also Lynch, Wagner, and many others) should be discussed here.

## References

[B1] Hartl DL, Clark AG (1997). Principles of Population Genetics.

[B2] Holland JH (1973). Genetic algorithms and the optimal allocation of trials. SIAM J Computing.

[B3] Holland JH (1975). Adaptation in Natural and Artificial Systems.

[B4] Goldberg DE (1989). Genetic Algorithms in Search, Optimization and Machine Learning.

[B5] Goldberg DE (2002). The Design of Innovation: Lessons From and For Competent Genetic Algorithms.

[B6] Koza JR, Bennett FH, Andre D, Keane MA (1999). Genetic Programming III: Darwinian Invention and Problem Solving.

[B7] Adami C (1997). Introduction to Artificial Life.

[B8] Lenski RE, Ofria C, Collier TC, Adami C (1999). Genome complexity, robustness and genetic interactions in digital organisms. Nature.

[B9] Wilke CO, Wang JL, Ofria C, Lenski RE, Adami C (2001). Evolution of digital organisms at high mutation rates leads to the survival of the flattest. Nature.

[B10] Lenski RE, Ofria C, Pennock RT, Adami C (2003). The evolutionary origin of complex features. Nature.

[B11] Chow SS, Wilke CO, Ofria C, Lensky RE, Adami C (2004). Adaptive radiation from resource competition in digital organisms. Science.

[B12] Francois P, Hakim V (2004). Design of genetic networks with specified functions by evolution in silico. Proc Natl Acad Sci USA.

[B13] Deckard A, Sauro HM (2004). Preliminary studies on the in silico evolution of biochemical networks. ChemBiochem.

[B14] Tyson JJ, Novak B (2001). Regulation of the eukaryotic cell cycle: molecular antagonism, hysteresis, and irreversible transitions. J Theor Biol.

[B15] Price ND, Reed JL, Palsson BO (2004). Genome-scale models of microbial cells: evaluating the consequences of constrains. Nat Rev Microbiol.

[B16] Ibarra RU, Edwards JS, Palsson BO (2002). Escherichia coli K-12 undergoes adaptive evolution to achieve in silico predicted optimal growth. Nature.

[B17] Mishchenko EF, Rozov NK (1980). Differential equations with small parameters and relaxation oscillations.

[B18] Crow JF, Kimura M (1979). Efficiency of truncation selection. Proc Natl Acad Sci USA.

[B19] Gilman A, Ross J (1995). Genetic-algorithm selection of a regulatory structure that directs flux in a simple metabolic model. Biophys J.

[B20] Bishop CM (1995). Neural Networks for Pattern Recognition.

[B21] Goebl MG, Petes TD (1986). Most of the yeast genomic sequences are not essential for cell growth and division. Cell.

[B22] Smith V, Chou KN, Lashkari D, Botstein D, Brown PO (1996). Functional analysis of the genes of yeast chromosome V by genetic footprinting. Science.

[B23] Thatcher JW, Shaw JM, Dickinson WJ (1998). Marginal fitness contributions of nonessential genes in yeast. Proc Natl Acad Sci USA.

[B24] Gerdes SY, Scholle MD, Campbell JW, Balazsi G, Ravasz E, Daugherty MD, Somera AL, Kyprides NC, Anderson I, Gelfand MS, Bhattacharya A, Kapatral V, D'Souza M, Baev MV, Grechkin Y, Mseeh F, Fonstein MY, Overbeek R, Barabasi AL, Oltvai ZN, Osterman AL (2003). Experimental determination and system level analysis of essential genes in Escherichia coli MG1655. J Bacteriol.

[B25] Kobayashi K, Ehrlich SD, Albertini A, Amati G, Andersen KK, Arnaud M, Asai K, Ashikaga S, Aymerich S, Bessieres P, Boland F, Brignell SC, Bron S, Bunai K, Chapuis J, Christiansen LC, Danchin A, Debarbouille M, Dervyn E, Deuerling E, Devine K, Devine SK, Dreesen O, Errington J, Fillinger S, Foster SJ, Fujita Y, Galizzi A, Gardan R, Eschevins C, Fukushima T, Haga K, Harwood CR, Hecker M, Hosoya D, Hullo MF, Kakeshita H, Karamata D, Kasahara Y, Kawamura F, Koga K, Koski P, Kuwana R, Imamura D, Ishimaru M, Ishikawa S, Ishio I, Le Coq D, Masson A, Mauel C, Meima R, Mellado RP, Moir A, Moriya S, Nagakawa E, Nanamiya H, Nakai S, Nygaard P, Ogura M, Ohanan T, O'Reilly M, O'Rourke M, Pragai Z, Pooley HM, Rapoport G, Rawlins JP, Rivas LA, Rivolta C, Sadaie A, Sadaie Y, Sarvas M, Sato T, Saxild HH, Scanlan E, Schumann W, Seegers JF, Sekiguchi J, Sekowska A, Seror SJ, Simon M, Stragier P, Studer R, Takamatsu H, Tanaka T, Takeuchi M, Thomaides HB, Vagner V, van Dijl JM, Watabe K, Wipat A, Yamamoto H, Yamamoto M, Yamamoto Y, Yamane K, Yata K, Yoshida K, Yoshikawa H, Zuber U, Ogasawara N (2003). Essential Bacillus subtilis genes. Proc Natl Acad Sci USA.

[B26] Sherr CJ, Roberts JM (2004). Living with or without cyclins and cyclin-dependent kinases. Genes Dev.

[B27] Gould SJ, Lewontin RC (1979). The spandrels of San Marco and the Panglossian paradigm: a critique of the adaptationist programme. Proc Royal Soc London Ser B.

[B28] Langdon WB, Poli R (2002). Foundations of Genetic Programming.

[B29] http://www.python.org.

[B30] http://www.activestate.com/Products/ActivePython/.

[B31] Press WH, Teukolsky SA, Vetterling WT, Flannery BP (2002). Numerical Recipes in C++.

[B32] http://www.swig.org.

